# Antitumor effects of the small molecule DMAMCL in neuroblastoma via suppressing aerobic glycolysis and targeting PFKL

**DOI:** 10.1186/s12935-021-02330-y

**Published:** 2021-11-24

**Authors:** Simeng Zhang, Zhongyan Hua, Gen Ba, Ning Xu, Jianing Miao, Guifeng Zhao, Wei Gong, Zhihui Liu, Carol J. Thiele, Zhijie Li

**Affiliations:** 1 Department of Pediatrics, Shengjing Hospital of China Medical University, Shenyang, China; 2 Medical Research Center, Liaoning Key Laboratory of Research and Application of Animal Models for Environment and Metabolic Diseases, Shengjing Hospital of China Medical University, #36 Sanhao Street, Heping District, Shenyang, 110004 China; 3 Cellular & Molecular Biology Section, Pediatric Oncology Branch, National Cancer Institute, National Institutes of Health Bethesda, Bethesda, MD 20892 USA

**Keywords:** Neuroblastoma, DMAMCL, Apoptosis, Aerobic glycolysis, PFKL

## Abstract

**Background:**

Neuroblastoma (NB) is a common solid malignancy in children that is associated with a poor prognosis. Although the novel small molecular compound Dimethylaminomicheliolide (DMAMCL) has been shown to induce cell death in some tumors, little is known about its role in NB.

**Methods:**

We examined the effect of DMAMCL on four NB cell lines (NPG, AS, KCNR, BE2). Cellular confluence, survival, apoptosis, and glycolysis were detected using Incucyte ZOOM, CCK-8 assays, Annexin V-PE/7-AAD flow cytometry, and Seahorse XFe96, respectively. Synergistic effects between agents were evaluated using CompuSyn and the effect of DMAMCL in vivo was evaluated using a xenograft mouse model. Phosphofructokinase-1, liver type (PFKL) expression was up- and down-regulated using overexpression plasmids or siRNA.

**Results:**

When administered as a single agent, DMAMCL decreased cell proliferation in a time- and dose-dependent manner, increased the percentage of cells in SubG1 phase, and induced apoptosis in vitro, as well as inhibiting tumor growth and prolonging survival in tumor-bearing mice (NGP, BE2) in vivo. In addition, DMAMCL exerted synergistic effects when combined with etoposide or cisplatin in vitro and displayed increased antitumor effects when combined with etoposide in vivo compared to either agent alone. Mechanistically, DMAMCL suppressed aerobic glycolysis by decreasing glucose consumption, lactate excretion, and ATP production, as well as reducing the expression of PFKL, a key glycolysis enzyme, in vitro and in vivo. Furthermore, PFKL overexpression attenuated DMAMCL-induced cell death, whereas PFKL silencing promoted NB cell death.

**Conclusions:**

The results of this study suggest that DMAMCL exerts antitumor effects on NB both in vitro and in vivo by suppressing aerobic glycolysis and that PFKL could be a potential target of DMAMCL in NB.

**Supplementary Information:**

The online version contains supplementary material available at 10.1186/s12935-021-02330-y.

## Background

Neuroblastoma (NB) is a prevalent childhood extracranial solid tumor that accounts for 7–8% of all pediatric malignancies and causes 15% of all pediatric oncology-related deaths [1, 2]. According to the International Neuroblastoma Pathology Classification, the pathological subtypes of neuroblastoma were divided into neuroblastoma (NB), ganglioneuroblastoma (GNB) and ganglioneuroma (GN). Compared with GNB and GN, NB had a lower degree of differentiation and a worse prognosis. Infants with high-risk NB tumors, typically those aged > 18 months with extensive metastasis, usually have a poor prognosis, with an overall survival rate of < 50% [3–5]. Consequently, more therapeutic strategies are urgently needed to improve the treatment efficacy for NB.

Micheliolide (MCL) is a natural guaianolide sesquiterpene lactone that is isolated from Michelia compressa and champaca plants [6, 7]. Dimethylaminomicheliolide (DMAMCL or ACT001) is a water-soluble adduct of MCL that releases MCL into plasma and allows good distribution in brain [8]. In addition, DMAMCL has a higher activity and stability in cells, lower toxicity, and fewer side-effects in animals than MCL [9, 10]. Studies have demonstrated that DMAMCL exerts good pharmacokinetic effects against adult tumors such as glioblastoma [11], breast cancer [12, 13], and colitis-associated cancer [14], indicating that DMAMCL could be a promising candidate for treating cancer. Notably, DMAMCL has recently been approved for phase I clinical trials in Australia for treating glioblastoma (Trial ID: ACTRN12616000228482) [15] and has been selected as an orphan agent by the Food and Drug Administration [16]; however, no studies have yet investigated the role of DMAMCL in NB and the mechanism of DMAMCL remains poorly understood.

Metabolic reprogramming has been shown to affect the survival of malignant tumor cells, with the most notable examples being the Warburg effect and aerobic glycolysis [17–19]. Glycolysis refers to the transformation of glucose to lactate when limited amounts of oxygen are available, known as anaerobic glycolysis in normal cells. However, Warburg defined the metabolism of cancer cells as aerobic glycolysis, underlining that the glucose to lactate conversion occurs regardless of whether oxygen is present. Even under normoxic conditions, most cancer cells consume glucose via glycolysis at a higher rate than normal cells [20, 21]; therefore, inhibiting aerobic glycolysis can suppress the growth of tumors, including NB [22–25]. However, it remains unclear whether aerobic glycolysis and related enzymes are involved in DMAMCL regulation.

In this study, we investigated the antitumor effects of DMAMCL as a single agent and combined with chemotherapeutic agents (etoposide and cisplatin) against NB in vitro and in vivo, as well as the underlying mechanisms involving aerobic glycolysis and PFKL, a rate-limiting glycolysis enzyme.

## Methods

### Cell culture and reagents

Four human NB cell lines (NGP, SK-N-AS [AS], SMS-KCNR [KCNR], SK-N-BE2C [BE2]) and a mouse fibroblast cell line (NIH3T3) were used in this study. NGP, BE2 and KCNR cells have *MYCN* amplification; AS, BE2, and KCNR cells have 1pLOH; AS and BE2 cells have P53 mutations; and KCNR cells has ALK mutation. All cell lines were received from Dr. Carol J. Thiele (National Institutes of Health, Bethesda, MD, USA) and tested mycoplasma contamination regularly. Cells were cultured in RPMI-1640 medium (Bioind, Kibbutz Beit Haemek, Israel) with 10% fetal bovine serum (Bioind), 2 mM glutamine (Bioind), and antibiotics (penicillin 100 units/mL, streptomycin 100 μg/mL; Bioind) at 37 °C in 5% CO_2_. DMAMCL (Nankai University, Tianjin, China) was a white powder with a molecular weight of 409.47. DMAMCL was dissolved in water to produce a 20 mM stock solution.

### Cell survival assays

To analyze cell survival, the four NB cell lines and NIH3T3 cell line were seeded onto 96-well plates, cultured overnight, and treated with different concentrations of DMAMCL (0–30 μM), etoposide (0–50 μg/mL), or cisplatin (0–20 μg/mL) for 72 h. Cell survival was then measured using a CCK-8 assay (Bimake, #B34304, China). Absorbance was detected at a wavelength of 450 nm using a microplate reader (Bio-Rad, Richmond, CA, USA).

### Incucyte ZOOM live cell imaging system

Cell confluence was evaluated in real time using Incucyte ZOOM (Essen BioSciences, Ann Arbor, MI, USA) every 4 h for 72 h. The cell confluence percentage was analyzed using Incucyte ZOOM software.

### Colony formation assay

Four NB cell lines and NIH3T3 (8 × 10^5^ (NGP, KCNR), 5 × 10^5^ (BE2), and 3 × 10^5^ (AS, NIH3T3)) were seeded onto 6 cm diameter plates respectively, and treated with different concentration of DMAMCL for 24 h, then the cells were collected, and 5 × 10^3^ (NGP, KCNR, BE2) or 2 × 10^3^ (AS, NIH3T3) viable cells were replated onto 6 cm diameter plates. After 14 days, visible colonies were fixed and stained for at least 1 h with Giemsa stain solution (Solarbio, #G1015, Beijing, China).

### Cell cycle assay

Four NB cell lines were seeded onto six-well plates, cultured overnight, and treated with different doses of DMAMCL for 48 h. The cells were collected, washed twice in cold phosphate-buffered saline (PBS), and fixed with cold 70% alcohol for 24 h. The cells were then incubated with 10 μg/mL RNase A (Beyotime, #C1052, China) and 50 μg/mL propidium iodide (Beyotime, #C1052, China) for 30 min in the dark at 37 °C. Stained cells were detected using flow cytometry (Becton Dickinson, Mountain View, CA, USA) and the percentage of cells at each phase of the cell cycle were analyzed using FlowJo software (Becton Dickinson).

### Apoptosis assay

Four NB cell lines were treated with different doses of DMAMCL for 48 h and then all cells and supernatants were harvested, washed twice with cold PBS, and resuspended with 100 μL 1× Annexin V binding solution. After the cells had been incubated with 5 μL of Annexin V-PE and/or 7-AAD (BD Biosciences, #559763, San Jose, CA, USA) at 37 °C in the dark for 15 min, 400 μL 1× Annexin V binding solution was added and apoptotic cells were detected using flow cytometry (Becton Dickinson). We added the cells at the early apoptotic stage (Annexin V+) and the cells at the late apoptotic stage (Annexin V+, 7-AAD+) together, defined them as the apoptotic cells, and then calculated the percent of apoptotic cells (apoptotic cells/total cells * 100%) for the comparison of the percent apoptotic cells among different groups.

### Western blotting

Protein lysates were extracted from NB cells or tumor tissues and subjected to sodium dodecyl sulfate-polyacrylamide gel electrophoresis (SDS-PAGE). The protein loading quantity was 30 μg. The separated proteins were transferred to Polyvinylidene fluoride (PVDF) membranes (Millipore, Bedford, MA, USA) which were probed with antibodies against cleaved-PARP (89 kDa, 1:1000, Cell Signaling Technology, #5625S, Beverly, MA, USA), HK2 (102 kDa, 1:1000, Cell Signaling Technology, #2867T), PFKL (78 kDa, 1:1000, Abcam, #ab181064, Cambridge, UK), PKM2 (60 kDa, 1:1000, Cell Signaling Technology, #4053T), and β tubulin (55 kDa, 1:2000, Proteintech, #10068-1-AP, Wuhan, China). Antibodies were detected using enhanced chemiluminescence (Thermo Scientific, Madison, WI, USA).

### Synergistic effect analysis

The combination effects between DMAMCL and chemotherapeutic agents (etoposide and cisplatin) in NGP and BE2 cells were evaluated using a CCK-8 assay. NGP cells were treated with DMAMCL (2.5, 3.75, 5 μM) and etoposide (0.02, 0.04, 0.05, 0.1, 0.2 μg/mL) or cisplatin (1, 2, 3 μg/mL) for 72 h. BE2 cells were treated with DMAMCL (2.5, 7.5, 10, 12.5 μM) and etoposide (0.05, 0.075, 0.1, 0.2 μg/mL) or cisplatin (0.5, 1, 3 μg/mL) for 72 h. Synergistic effects were analyzed using CompuSyn software (CompuSyn, Inc., Paramus, NJ, USA). CI values of < 1 indicate synergism, CI = 1 reflects an additive effect, and CI > 1 indicates antagonism.

### Glycolysis measurements

To assess glycolytic function, we used an Agilent Seahorse XF Glycolysis Stress Test Kit (Seahorse Bioscience, #16814032, North Billerica, MA, USA) with a Seahorse XFe96 Analyzer to directly measure the real time extracellular acidification rate (ECAR). Before measurement, the sensor cartridge was hydrated in Seahorse XF Calibrant at 37 °C in a non-CO_2_ incubator overnight. NGP, BE2 and NIH3T3 cells were seeded onto 6-well plates with different doses of DMAMCL, incubated for 24 h, and then the cells were collected, washed twice with cold PBS, and resuspended with phenol red-free assay solution. To avoid the impact of the different number of cells in each group on ECAR, we counted and seeded the same number of cells for each group onto the XF96 cell culture microplates (Seahorse Bioscience, North Billerica, MA, USA) precoated with Cell-Tak solution at the density of 4 × 10^4^ cells/well (NGP) and 3.5 × 10^4^ cells/well (BE2, NIH3T3). The cell culture microplates were centrifuged at 1000 rpm for 5 min to attach the cells and then glucose, oligomycin (ATP synthase inhibitors), and 2-deoxy-d-glucose (2-DG, hexokinase inhibitor) were added according to the manufacturer’s instructions (Seahorse Bioscience) and detected immediately.

### Extracellular glucose concentration, lactate excretion, and ATP production assays

Extracellular glucose concentration and lactate production were determined using a Glucose Assay Kit (Biovision, Mountain View, #K606-100, CA, USA) and Lactate Assay Kit (Biovision, #K607-100) respectively. ATP levels were analyzed using an enhanced ATP Assay Kit (Beyotime, #S0027, China). Briefly, cells were incubated in 6-well plates overnight and then treated with DMAMCL for 24 h. The supernatants were collected to detect extracellular glucose concentration and lactate production, while the cells were collected and lysed to detect ATP levels. Cell protein concentration was also measured to eliminate errors because of differences in protein content.

### Cell transfection

PFKL overexpression plasmids (GeneCopoeia, Guangzhou, China) and small interfering RNAs (siRNA #1, #2, #3; Wanze, Anhui, China) were designed to up- and down-regulate PFKL expression in NB cells, respectively. The following siRNA target sequences were used: PFKL #1, 5′-CGG AGA UGA AGA CAG ATT-3′; PFKL #2, 5′-UCU GUC UUC AUC UUC UCC GTT-3′; PFKL #3, 5′-CCA CGG AGU UCC UGU ACA ATT-3′; control, 5′-UUC UCC GAA CGU GUC ACG UTT-3′. NB cells were transfected with the plasmids and siRNAs using jetPRIME agent (Polyplus Transfection, #114-15, Illkirsch, France) for 16 h and then treated with DMAMCL before further experiments.

### TUNEL assay

Paraffin-embedded tumor tissue slides were prepared from the in vivo experiments and apoptosis was detected using a TUNEL staining kit (Roche Molecular Biochemicals, #11684817910, Indianapolis, IN, USA), according to the manufacturer’s instructions. TUNEL-positive cells were observed under a fluorescence microscope (Olympus, Tokyo, Japan). The magnification of the images was 200×, Scale bar: 100 μm.

### In vivo* experiments*

The right flanks of 4- to 6-week-old female BALB/C nude mice (Beijing Huafukang Bioscience, Beijing, China) were subcutaneously inoculated with 100 μL of cell suspension containing 3 × 10^6^ cells. When the tumors had reached 100–150 mm^3^, DMAMCL (75 mg/kg, or 100 mg/kg) or a placebo was administered once daily for up to 21 days by oral gavage (*n* = 10 per group). Etoposide (15 mg/kg) was administered once a week for up to 3 weeks via intraperitoneal injection (*n* = 10 per group) [26, 27]. The tumors were measured three times a week and the tumor volume was calculated as $\frac{L \times {W}^{2}}{2}$, where L = length (mm) and W = width (mm). Mouse survival was measured from the beginning of treatment to the end of the experiment, which is when the tumor size reached 20 mm or the mouse had to be euthanized due to the poor general condition. For cleaved-PARP and PFKL expression analysis and terminal deoxynucleotidyl transferase using peroxide12-UTP nick end labeling (TUNEL) staining, mice bearing NGP and BE2 xenograft tumors were treated with DMAMCL (75 mg/kg, 100 mg/kg) for 1 week and tumor tissues were harvested (*n* = 3 per group). At end of the experiment, the mice were euthanized as follows: the carbon dioxide (CO_2_) was given with a flow rate of 20% chamber vol/min for 2–3 min when the mice were insensible, then gradually increased to 100% chamber vol/min till the mice were confirmed dead after 5 min of apnea. All mice were housed at 23 ± 2 °C with 40–70% humidity. All animal experiments followed ethical standards and were approved by the Experimental Animal Ethical Committee of Shengjing Hospital of China Medical University (2019PS302K).

### Statistical analysis

In vitro and in vivo data were presented as the mean ± standard deviation (SD) and the mean ± standard error (SE), respectively. Mouse survival was calculated using the Log-Rank test. Statistical analysis was performed using GraphPad Prism 8 software version 8.2.1 (GraphPad Software, Inc., La Jolla, CA, USA). Between-group comparisons were made using unpaired Student’s *t* tests. A *p* value < 0.05 were considered statistically significant, and *p < 0.05; **p < 0.01; ***p < 0.001.

## Results

### DMAMCL induces NB cell death via apoptosis

**Fig. 1:**
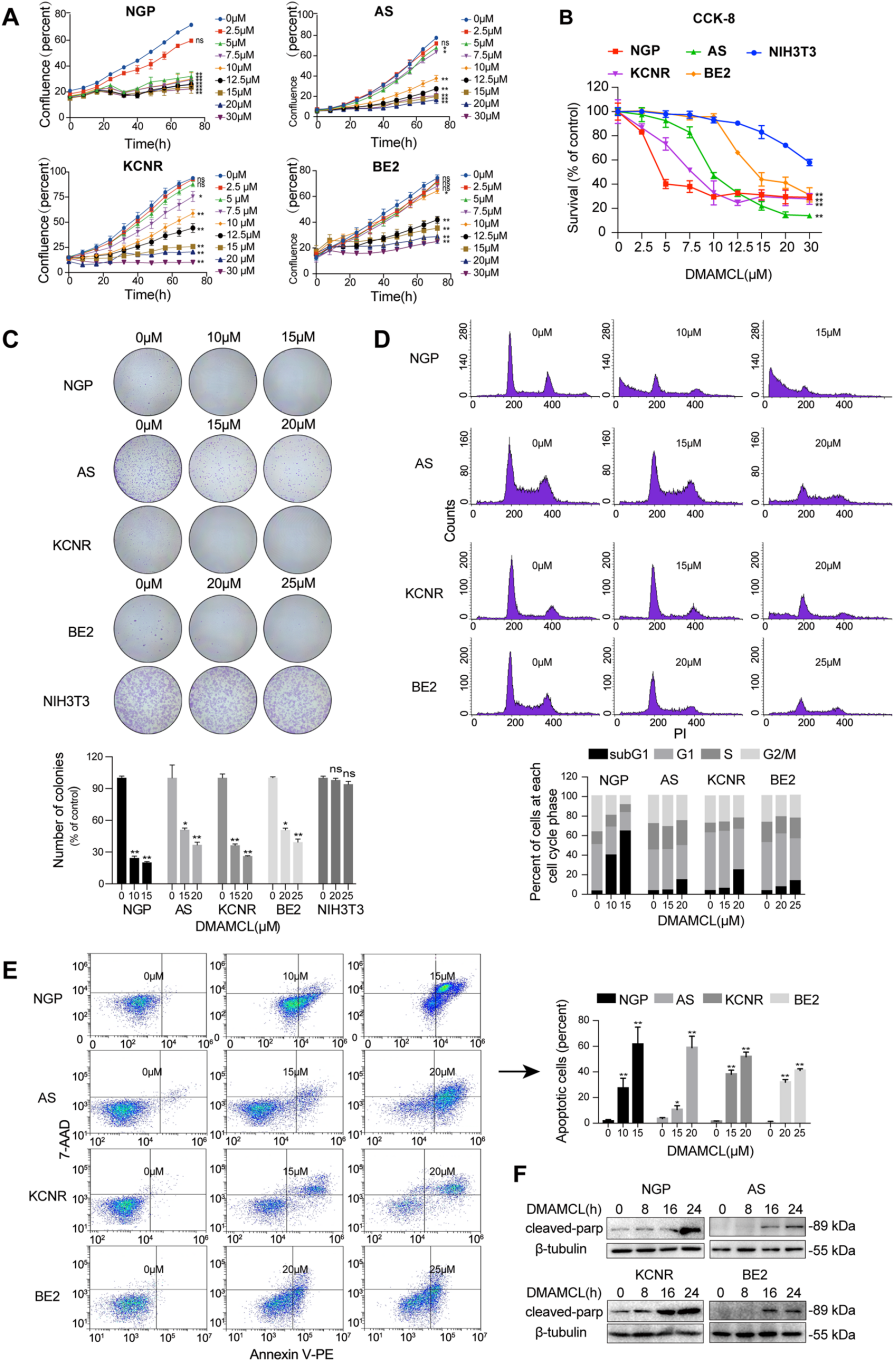
DMAMCL induced NB cell apoptosis in vitro. Four NB cell lines (NGP, AS, KCNR, BE2) and NIH3T3 cells were treated with different DMAMCL concentrations. **A** Cell confluence was monitored dynamically using Incucyte ZOOM for 72 h, ns (not significant): control vs DMAMCL treatment, *p < 0.05; **p < 0.01; ***p < 0.001. **B** Cell survival was detected using a CCK-8 assay at 72 h. **C** Colony formation assays were performed in the four NB cells and NIH3T3. Data were presented as mean ± SD. **D** Cell cycle was analyzed by detecting the percentage of PI-staining cells at each stage using flow cytometry at 48 h treatment of DMAMCL. **E** Cell apoptosis was detected using Annexin V-PE/7-AAD flow cytometry at 48 h. Data represent the mean ± SD of three independent experiments. **F** Cleaved PARP expression was detected using western blotting after DMAMCL treatment for 0, 8, 16 and 24 h

To investigate the effect of DMAMCL in NB cells, we treated four NB cell lines (NGP, AS, KCNR and BE2) with different concentrations of DMAMCL. Cell confluence was dynamically monitored using Incucyte every 4 h for 72 h (Fig. 1A) and cell survival was measured using CCK-8 assays at 72 h (Fig. 1B). We found that DMAMCL treatment decreased cell confluence in a time- and dose-dependent manner, indicating that DMAMCL inhibits cell proliferation (Fig. 1A). DMAMCL also dose-dependently inhibited NB cell survival compared to the control NIH3T3 cells (IC_50_ > 30 μM vs. 3.2 μM for NGP, 9.5 μM for AS, 7.0 μM for KCNR, and 13.0 μM for BE2; Fig. 1B). Colony formation assay also indicated that the colony-forming ability of the four NB cells with DMAMCL pretreatment for 24 h was significantly reduced compared to the control group, while no significant difference was observed in NIH3T3 cells (Fig. 1C). Among the four NB cell lines, NGP cells was the most sensitive to DMAMCL treatment, while BE2 cells was the least sensitive.

To explore whether DMAMCL induced NB cell death via apoptosis, we treated the four NB cell lines with different concentrations of DMAMCL for 48 h and then stained the cells with PI for cell cycle analysis (Fig. 1D) or Annexin V-PE/7-AAD for apoptosis analysis (Fig. 1E) by flow cytometry. We observed a significant increase in the proportion of SubG1 phase cells after DMAMCL treatment in all four NB cell lines, particularly at higher DMAMCL concentrations (Fig. 1D). Similarly, Annexin V-PE/7-AAD flow cytometry revealed a significant increase in the proportion of apoptotic cells in all four NB cell lines after DMAMCL treatment (Fig. 1E). We also detected the expression of cleaved-PARP, a marker of apoptosis, in the DMAMCL-treated NB cell lines, finding that DMAMCL increased cleaved-PARP expression in a time-dependent manner (Fig. 1F). Together, these data indicate that DMAMCL induces NB cell death via apoptosis.

### DMAMCL exerts antitumor effects in vivo

**Fig. 2:**
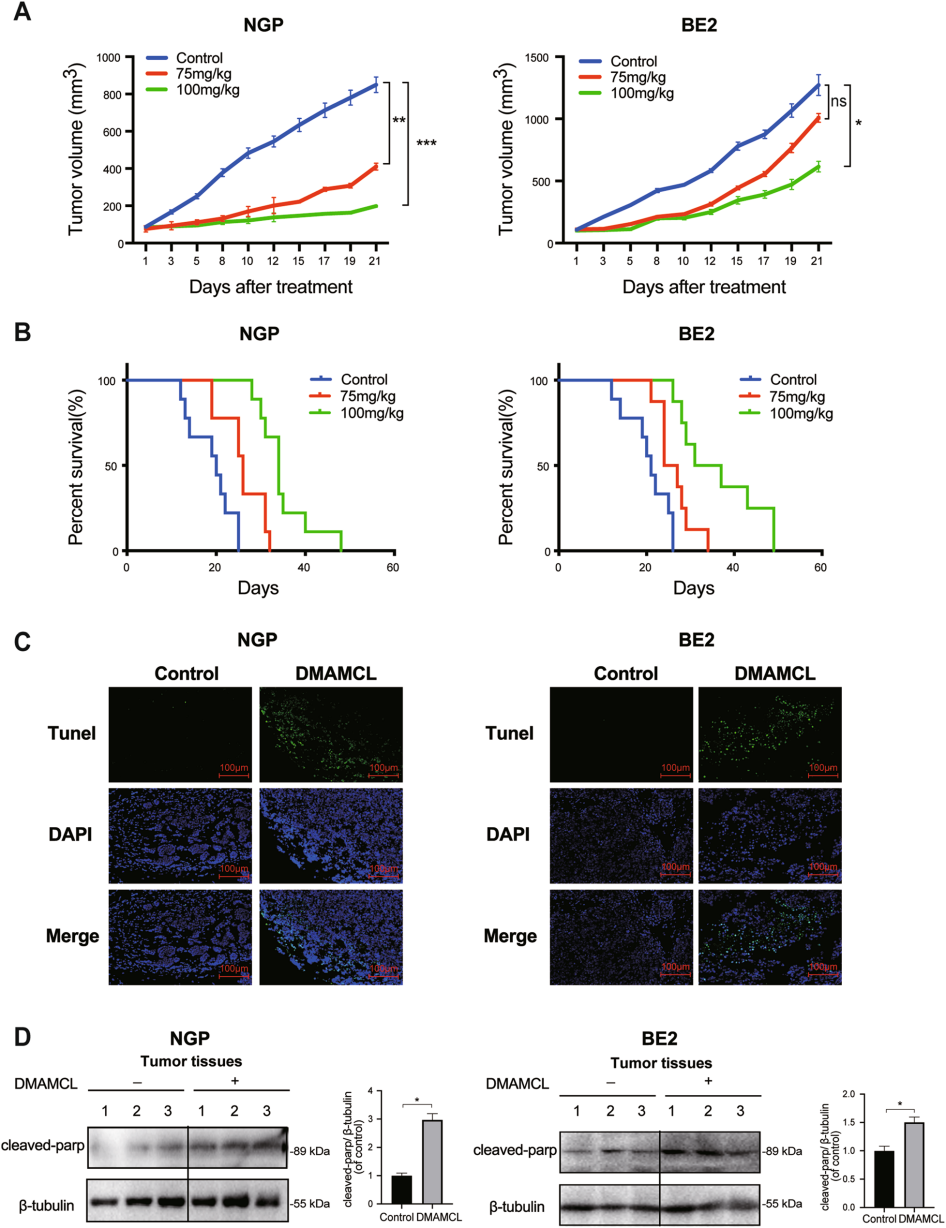
DMAMCL inhibited tumor growth and prolonged survival in mice bearing NB tumors. BALB/C nude mice were inoculated with NGP and BE2 cells and then treated with DMAMCL (75 or 100 mg/kg) for 21 days. **A** Tumor volume was measured three times a week and compared at the end of the experiment. Error bar: SE, ns (not significant): control vs DMAMCL treatment, *p < 0.05; **p < 0.01; ***p < 0.001; NGP (*n* = 10), BE2 (*n* = 10). **B** Survival curves of mice bearing NGP and BE2 tumors plotted using Kaplan–Meier analysis. **C** Representative TUNEL staining images (green) of tumor tissue from mice bearing NGP and BE2 tumors after DMAMCL (75 or 100 mg/kg) treatment for a week. Nuclei were stained with DAPI (blue). The magnification of the images was 200×. Scale bar: 100 μm. **D** Cleaved PARP expression in NGP and BE2 tumor tissues detected by western blotting, *p < 0.05; **p < 0.01; ***p < 0.001

To determine whether DMAMCL treatment affected NB tumor growth in vivo, we treated mice bearing NGP and BE2 xenograft tumors with DMAMCL (75 mg/kg, 100 mg/kg) (Additional file 1: Fig. S1). Interestingly, DMAMCL inhibited the growth of NGP tumors by 51.6% (75 mg/kg, *p* < 0.01) and 76.6% (100 mg/kg, *p* < 0.001) compared to control group and inhibited the growth of BE2 tumors by 20.7% (75 mg/kg, *p* = 0.38) and 51.5% (100 mg/kg, *p* < 0.05; Fig. 2A). These data showed that BE2 xenograft tumors were less sensitive to DMAMCL compared to NGP xenograft tumors.

Next, we evaluated the survival of NGP and BE2 tumor-bearing mice after DMAMCL treatment using Kaplan–Meier survival curves, observing a significant survival advantage in DMAMCL-treated mice compared to the control groups (Fig. 2B). In NGP tumor-bearing mice, the median survival time was 26 (75 mg/kg, *p* < 0.01) or 34 (100 mg/kg, *p* < 0.001) days in the DMAMCL-treated groups compared to 20 days in the control group. Similarly, the median survival time of BE2 tumor-bearing mice was 25.5 (75 mg/kg, *p* < 0.05) or 34 (100 mg/kg, *p* < 0.001) days in the DMAMCL-treated groups compared to 22 days in the control group.

To confirm that DMAMCL induced apoptosis in NB tumors in vivo, we harvested NGP and BE2 xenograft tumor tissues from mice treated with DMAMCL for a week and performed TUNEL staining and detected cleaved-PARP protein expression. As expected, more TUNEL-positive cells (green) were observed in the NGP and BE2 tumors after DMAMCL treatment (Fig. 2C). Furthermore, densitometry analysis revealed that cleaved-PARP expression was significantly higher in DMAMCL-treated NGP or BE2 xenograft tumors than in the control group (*p* < 0.05). Therefore, these data indicate that DMAMCL exerts antitumor growth effects and extends the survival of mice bearing NB xenograft tumors.

### DMAMCL exerts synergistic effects with chemotherapeutic agents in vitro

**Fig. 3:**
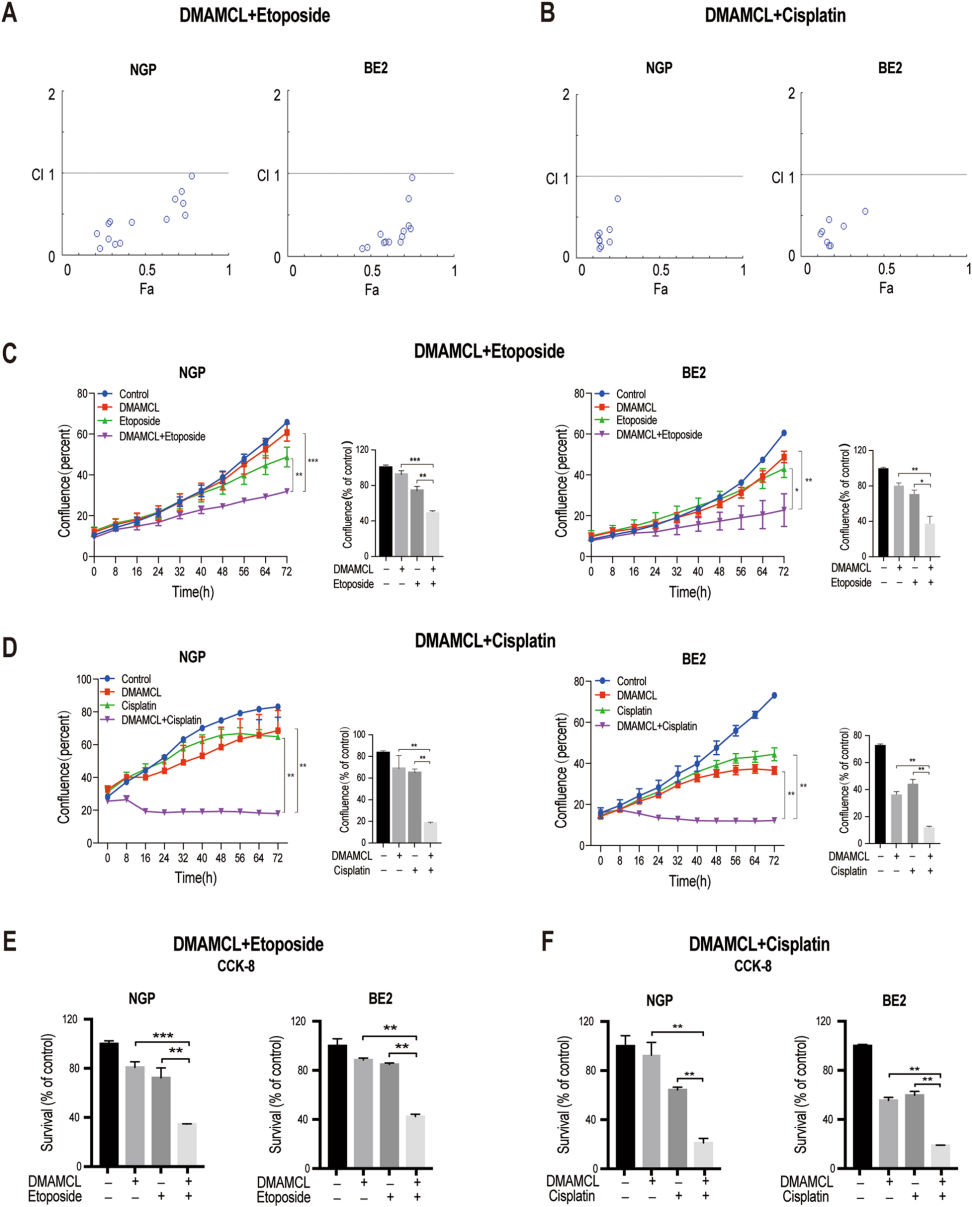
Synergistic effects of DMAMCL with etoposide or cisplatin in NB cells. NGP and BE2 cells were treated with different concentrations of DMAMCL with etoposide or cisplatin alone or in combination for 72 h. The combination index (CI) was analyzed using CompuSyn. CI < 1 indicates synergism, CI = 1 reflects an additive effect, and CI > 1 indicates antagonism. Cell confluence and survival were detected using Incucyte ZOOM and CCK-8 assays, respectively. **A** CI values of NGP and BE2 cells treated with the combination of DMAMCL and etoposide. **B** CI values of NGP and BE2 cells treated with the combination of DMAMCL and cisplatin. **C** Confluence of NGP and BE2 cells treated with the indicated concentration of DMAMCL and etoposide alone or in combination, *p < 0.05; **p < 0.01; ***p < 0.001. **D** Confluence of NGP and BE2 cells treated with the indicated concentration of DMAMCL and cisplatin alone or in combination. **E** Survival of NGP and BE2 cells treated with the indicated concentration of DMAMCL and etoposide alone or in combination. **F** Survival of NGP and BE2 cells treated with the indicated concentration of DMAMCL and cisplatin alone or in combination

To investigate the combination effects between DMAMCL and the chemotherapeutic agents etoposide and cisplatin, we treated NGP and BE2 cells with DMAMCL alone or in combination with these agents and detected cell survival using CCK-8 assays at 72 h (Additional file 2: Fig. S2). The synergistic effects between DMAMCL and etoposide/cisplatin were evaluated by calculating the combination index (CI) value using CompuSyn. We observed a synergistic effect (CI < 1) between DMAMCL and etoposide in NGP (Fig. 3A, left) and BE2 (Fig. 3A, right) cells, with similar results when DMAMCL was combined with cisplatin (Fig. 3B).

Therefore, we treated NGP and BE2 cells with representative concentrations of DMAMCL, etoposide, and cisplatin individually or in combination and detected cell confluence using Incucyte every 4 h for 72 h. Interestingly, the confluence of NGP cells treated with DMAMCL + etoposide (48.4%) was significantly lower than that of cells treated with either agent alone (92.3 and 74.1%, respectively; Fig. 3C, left), indicating that the combination inhibited cell growth to a greater degree. Similar results were also observed in BE2 cells treated with DMAMCL + etoposide (Fig. 3C, right) and in NGP and BE2 cells treated with DMAMCL + cisplatin (Fig. 3D).

In addition, we analyzed the survival of NGP and BE2 cells treated with the same concentrations of DMAMCL, etoposide, and cisplatin individually or in combination. We found that the survival of NGP cells treated with the combination (DMAMCL + etoposide) was significantly lower (34.6%) than when treated with either DMAMCL (80.8%, *p* < 0.001) or etoposide (72.3%, *p* < 0.01; Fig. 3E, left). Similar trends were found in BE2 cells treated with DMAMCL + etoposide (Fig. 3E, right) and in NGP and BE2 cells treated with DMAMCL + cisplatin (Fig. 3F), consistent with the observed changes in cell confluence. Together, these data indicate that DMAMCL exerts synergistic effects against NB cell growth in combination with etoposide or cisplatin.

### The combination of DMAMCL and etoposide exert increased antitumor growth effects in vivo

**Fig. 4:**
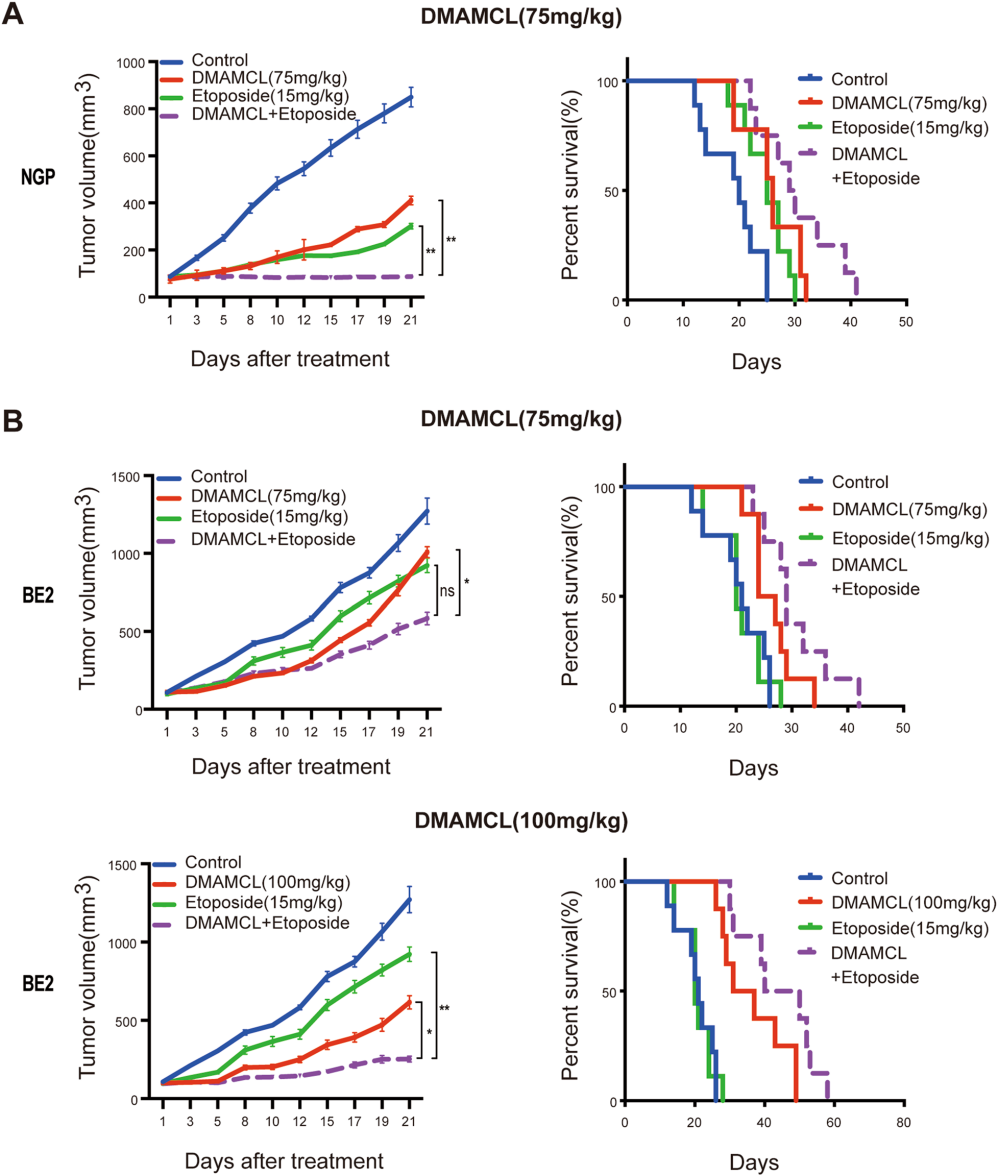
The combination of DMAMCL and etoposide increased antitumor effects and prolonged the survival of mice compared to either agent alone. Xenograft mouse models were established as described in Fig. 2. NGP tumor-bearing mice were treated with DMAMCL (75 mg/kg) or etoposide (15 mg/kg) alone or in combination for 21 days. BE2 tumor-bearing mice were treated with DMAMCL (75 or 100 mg/kg) or etoposide (15 mg/kg) alone or in combination for 21 days. **A** Tumor volume and survival curves of NGP tumor-bearing mice. Error bar: SE, ns (not significant): DMAMCL/Etoposide vs DMAMCL + Etoposide, *p < 0.05; **p < 0.01; ***p < 0.001, NGP (*n* = 10). **B** Tumor volume and survival curves of BE2 tumor-bearing mice. Error bar: SE, ns (not significant): DMAMCL/Etoposide vs DMAMCL + Etoposide, *p < 0.05; **p < 0.01; ***p < 0.001, BE2 (*n* = 10)

To confirm the combination effects of DMAMCL and etoposide in vivo, we treated NB xenograft tumor-bearing mice with DMAMCL and etoposide, either alone or in combination. Since DMAMCL inhibited NGP xenograft tumor growth by 77% at 100 mg/kg (Fig. 2A), we used 75 mg/kg DMAMCL for the DMAMCL + etoposide (15 mg/kg) combination in these experiments (Fig. 4A) but both doses of DMAMCL (75 and 100 mg/kg) in BE2 tumors (Fig. 4B).

After 21 days of treatment, DMAMCL + etoposide had completely inhibited the growth of NGP tumors, thereby significantly reducing tumor growth compared to either agent alone (*p* < 0.01, Fig. 4A, left). In addition, DMAMCL + etoposide treatment produced a significant survival advantage in mice bearing NGP tumors, increasing the median survival time to 30 days compared to 26 days in the DMAMCL group and 25 days in the etoposide group (Fig. 4A, right).

In BE2 tumor-bearing mice, DMAMCL(75 mg/kg) + etoposide inhibited tumor growth by 54%, which was significantly higher than either DMAMCL (21%, *p* < 0.05) or etoposide (27%, *p* = 0.08) alone. Moreover, the DMAMCL(75 mg/kg) + etoposide group displayed a significantly longer median survival time (29 days) than the DMAMCL (24 days, *p* = 0.13) or etoposide (21 days, *p* < 0.01) groups (Fig. 4B, upper). Similar results were observed for the DMAMCL(100 mg/kg) + etoposide combination (Fig. 4B, lower), indicating that the combination of DMAMCL and etoposide exerts a significantly greater effect against tumor growth than either individual agent in mice bearing NB xenograft tumors.

### DMAMCL suppresses aerobic glycolysis and PFKL expression in NB cells

**Fig. 5:**
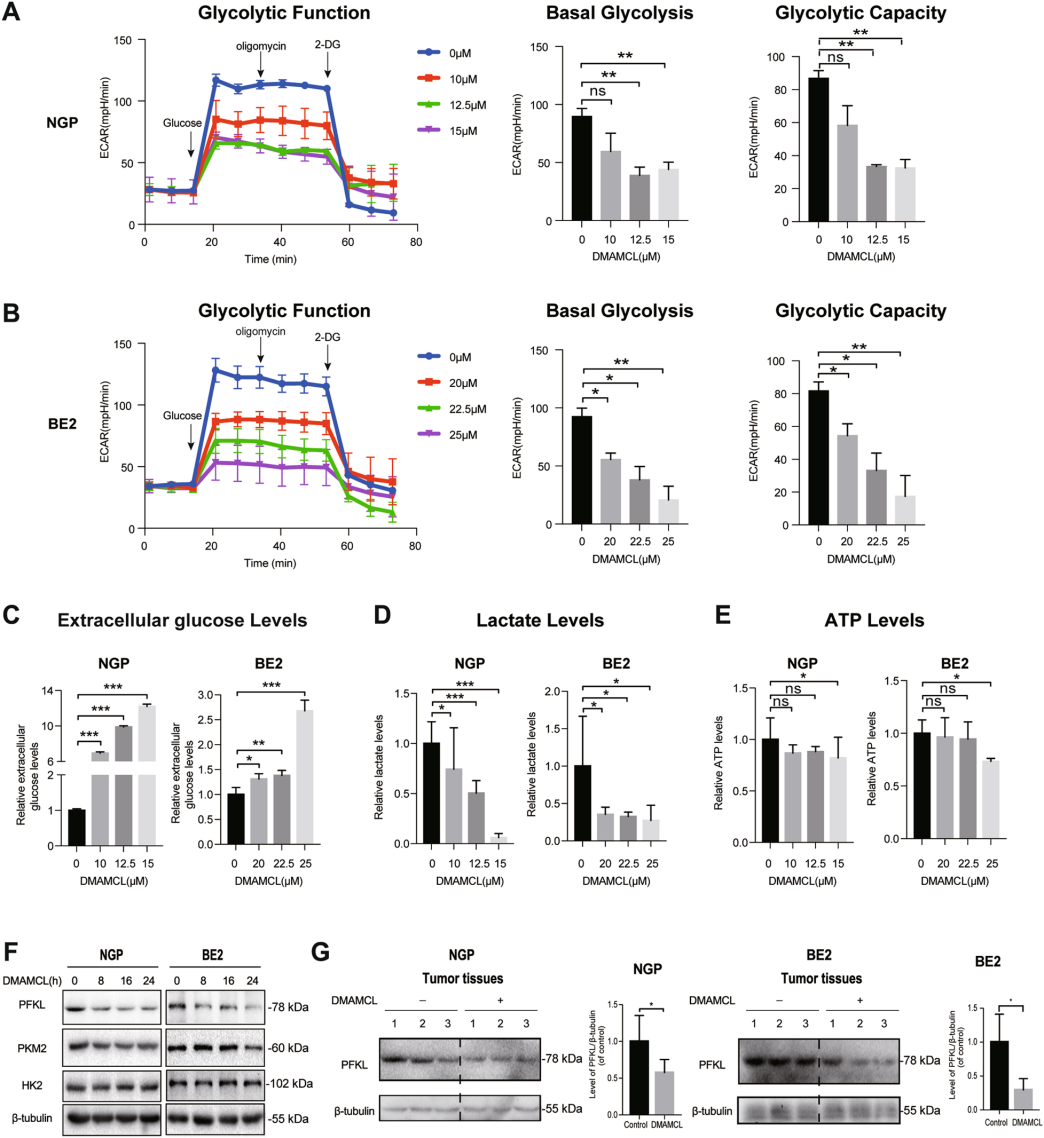
DMAMCL suppressed aerobic glycolysis and decreased PFKL expression level in NB. After NGP and BE2 cells were treated with different DMAMCL concentrations for 24 h, ECAR was measured by adding glucose, oligomycin (ATP synthase inhibitors), and 2-deoxy-d-glucose (2-DG, hexokinase inhibitor) in turn to reflect the glycolysis rate including the glycolytic function, basal glycolysis, and glycolytic capacity, while extracellular glucose, extracellular lactate, and intracellular ATP levels were also measured. Glycolytic function, basal glycolysis, and glycolytic capacity of **A** NGP cells and **B** BE2 cells, ns (not significant): control vs DMAMCL treatment, *p < 0.05; **p < 0.01; ***p < 0.001. **C** Extracellular glucose concentration, **D** lactate excretion, and **E** intracellular ATP levels of NGP and BE2 cells. **F** PFKL, PKM2, and HK2 expression in NGP and BE2 cells after DMAMCL treatment for 0, 8, 16, and 24 h measured using western blotting. **G** PFKL expression in NGP and BE2 tumor tissues after DMAMCL treatment. *p < 0.05; **p < 0.01; ***p < 0.001

To evaluate the potential mechanism underlying DMAMCL-induced cell death, we examined the effect of different DMAMCL concentrations on aerobic glycolysis in NGP and BE2 cells. The extracellular acidification rate (ECAR) was analyzed after the serial addition of glucose, oligomycin, and 2-deoxyglucose. In NGP cells, different concentrations of DMAMCL significantly decreased the ECAR compared to cells in the control group, suggesting that DMAMCL reduced the rate of glycolysis (Fig. 5A, left). Moreover, higher concentrations of DMAMCL substantially decreased basal glycolysis (Fig. 5A, middle) and glycolytic capacity (Fig. 5A, right), with similar results observed in BE2 cells (Fig. 5B). Besides, we also detected the glycolysis of NIH3T3 after DMAMCL (0, 15, 20, 25 μM) treatment and found that the basal glycolysis and glycolytic capacity were slightly reduced in DMAMCL-treated group, but there was no statistical difference compared to the control group (Additional file 3: Fig. S3).

We also evaluated extracellular glucose and lactate levels in cell culture supernatants and intracellular ATP levels in NGP and BE2 cells after DMAMCL treatment. Consistently, DMAMCL increased extracellular glucose content (Fig. 5C), indicating that glucose consumption was inhibited in NB cells, but decreased extracellular lactate content, indicating that glycolysis was inhibited (Fig. 5D). Furthermore, DMAMCL decreased intracellular ATP levels in NB cells (Fig. 5E), indicating a decrease in energy production. Together, these results are consistent with the inhibition of aerobic glycolysis detected using the Seahorse XFe96 Analyzer (Fig. 5A, B).

Having found that DMAMCL treatment inhibited aerobic glycolysis in NB cells (Fig. 5), we decided to investigate whether key glucose metabolism enzymes (PFKL, PKM2, and HK2) were involved in the effects of DMAMCL by evaluating their protein expression in NB cells after DMAMCL treatment (8, 16, or 24 h). Interestingly, PFKL expression was reduced after 8 h of DMAMCL treatment and continued to decrease until 24 h after treatment in both NGP and BE2 cells (Fig. 5F), whereas PKM2 and HK2 protein expression only decreased after 24 h of DMAMCL treatment. In addition, we found that PFKL expression was significantly lower in NGP and BE2 xenograft tumor-bearing mice treated with DMAMCL than in the control groups (Fig. 5G). Therefore, DMAMCL appears to suppress aerobic glycolysis and decrease PFKL expression in NB.

### PFKL overexpression blocks DMAMCL-induced NB cell death

Since DMAMCL treatment decreased PFKL expression in NB cells, we investigated whether PFKL overexpression affected DMAMCL-induced NB cell death by transfecting NGP and BE2 cells with PFKL overexpression plasmids to up-regulate PFKL. After the cells had been treated with DMAMCL, cell confluence, survival, and apoptosis were assayed as described in Fig. 1.

**Fig. 6:**
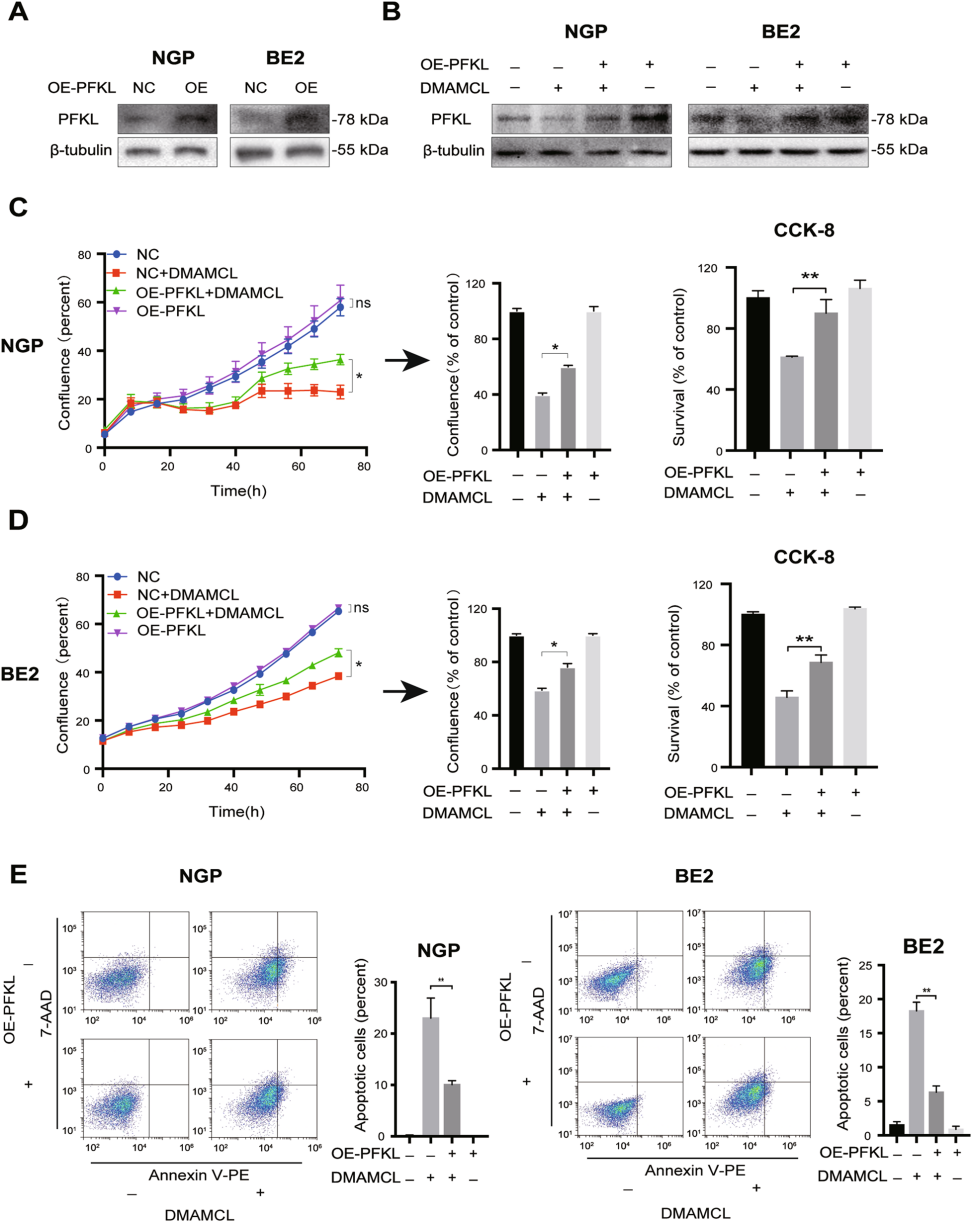
PFKL overexpression partially blocked DMAMCL-induced NB cell death. NGP and BE2 cells were transfected with PFKL overexpression or control plasmids for 16 h before being treated with DMAMCL for 72 h. Cell confluence, survival, and apoptosis were detected as described in Fig. 1. **A** PFKL expression in NGP and BE2 cells transfected with PFKL overexpression or control plasmids detected using western blotting. **B** PFKL expression in NGP and BE2 cells treated with DMAMCL + PFKL overexpression or control plasmids. **C** Confluence and survival of NGP cells treated with DMAMCL + PFKL overexpression or control plasmids, ns (not significant): NC vs OE-PFKL, *p < 0.05; **p < 0.01; ***p < 0.001. **D** Confluence and survival of BE2 cells treated with DMAMCL + PFKL overexpression or control plasmids. **E** Proportion of apoptotic NGP and BE2 cells treated with DMAMCL + PFKL overexpression or control plasmids. Data represent the mean ± SD of three independent experiments, *p < 0.05; **p < 0.01; ***p < 0.001

As expected, PFKL protein expression was significantly higher in NGP and BE2 cells transfected with the PFKL overexpression plasmid (Fig. 6A) and attenuated the DMAMCL-induced decrease in PFKL (Fig. 6B). In NGP cells, confluence was similar when transfected with the PFKL overexpression or control plasmids; however, DMAMCL significantly increased the confluence of cells overexpressing PFKL (59.8%) compared to the control cells (39.6%, *p* < 0.05; Fig. 6C, left and middle), as well as cell survival (84.7% vs. 61.2%, *p* < 0.01; Fig. 6C, right). Similar results were also observed in BE2 cells treated under the same conditions (Fig. 6D). Annexin V-PE/7-AAD flow cytometry revealed that there were significantly fewer apoptotic PFKL-overexpressing NGP cells when treated with DMAMCL (10.1%) compared to the control (23.0%, *p* < 0.01, Fig. 6E, left). Consistently, similar results were obtained in BE2 cells transfected with PFKL overexpression/control plasmids and treated with DMAMCL (Fig. 6E, right). Thus, these data indicate that PFKL up-regulation partially blocks DMAMCL-induced cell death in NB cells.

### PFKL down-regulation promotes NB cell death

**Fig. 7:**
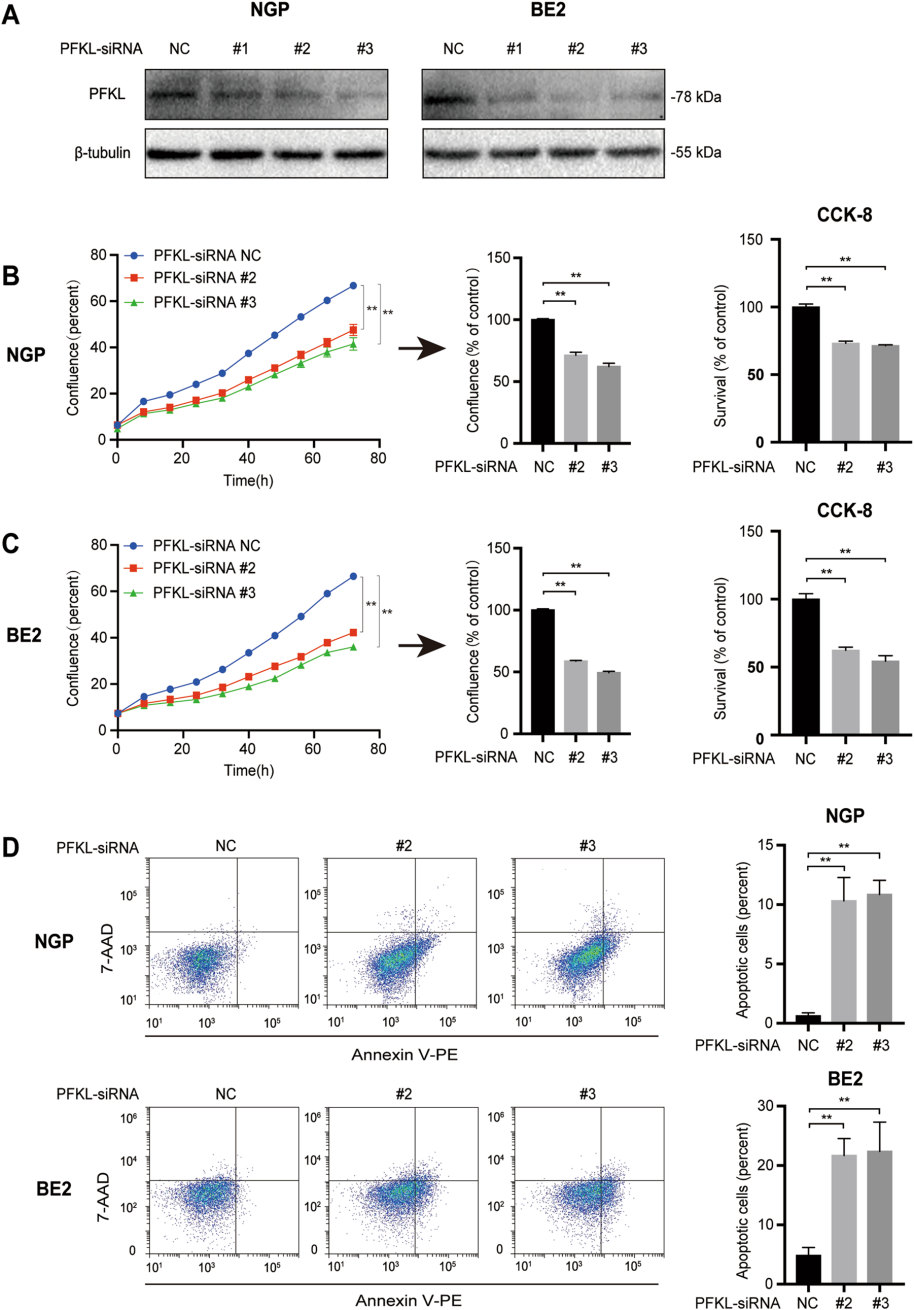
PFKL down-regulation induced NB cell death. NGP and BE2 cells were transfected with PFKL or control siRNAs for 16 h. Cell confluence, survival, and apoptosis were detected as described in Fig. 1. **A** PFKL expression in NGP and BE2 cells transfected with PFKL and control siRNAs. **B**, **C** Confluence and survival of **B** NGP cells and **C** BE2 cells transfected with PFKL and control siRNAs, *p < 0.05; **p < 0.01; ***p < 0.001. **D** Proportion of apoptotic NGP and BE2 cells transfected with PFKL or control siRNAs. Data represent the mean ± SD of three independent experiments, *p < 0.05; **p < 0.01; ***p < 0.001

To further elucidate the effect of PFKL on NB cell death, we designed three PFKL siRNAs (#1, #2, #3) to down-regulate endogenous PFKL expression in NGP and BE2 cells (Fig. 7A). PFKL siRNAs #2 and #3 were used for subsequent experiments assaying cell confluence, survival, and apoptosis.

Confluence curves revealed that confluence was lower in NGP cells transfected with PFKL siRNA than in those transfected with control siRNA (Fig. 7B, left). In particular, confluence was significantly lower in NGP cells 72 h after transfection with PFKL siRNA #2 (71.2%) and PFKL siRNA #3 (62.1%) than with control siRNA (set at 100%, *p* < 0.01; Fig. 7B, middle). Consistently, cell survival was lower for cells transfected with PFKL siRNA #2 or siRNA #3 (73.1% or 71.4%, respectively) compared to control siRNA (set at 100%, *p* < 0.01; Fig. 7B, right), whereas the percentage of apoptotic cells was significantly higher (10.3% and 10.8% vs. 0.6%, *p* < 0.01; Fig. 7D, upper). Similar results were observed when BE2 cells were transfected with PFKL or control siRNAs (Fig. 7C, D, lower). Together, these data indicate that downregulating PFKL expression enhances NB cell death.

## Discussion

DMAMCL has been shown to exert inhibitory effects against various cancers, including glioma [26], hepatocellular carcinoma (HCC) [27], leukemia [28, 29], and breast cancer [30], when administered as a single agent. In this study, we found that DMAMCL could exert antitumor growth effects against NB in vitro and in vivo both when administered alone or in combination with chemotherapeutic agents. In addition, we demonstrated that PFKL mediates DMAMCL-induced NB cell death by affecting aerobic glycolysis.

As a naturally derived anticancer drug, DMAMCL has received increasing attention owing to its minimal toxicity. Apoptosis is a programmed cell death mechanism that plays an essential role in cancer development. Recent studies have demonstrated that DMAMCL induces apoptosis in gliomas and gastric cancer cells [26, 31]. In this study, DMAMCL induced NB cell death via apoptosis but had much lower toxicity in control NIH3T3 cells. Consistent results were observed in four different NB cell lines, representative of the heterogeneity typically found in NB tumors and cell lines, which suggests that DMAMCL has a universal inhibiting effect on different types of NB tumors. Moreover, we found that DMAMCL suppressed NB tumor growth and improved survival in a xenograft mouse model without significantly affecting body weight (Additional file 4: Fig. S4A), consistent with a previous study in which DMAMCL suppressed tumor growth in a mouse xenograft model of HCC [27]. However, although DMAMCL had inhibitory effects on different NB cell lines in vitro and in vivo, their sensitivity to DMAMCL was also different. Among them, BE2 had the least sensitivity to DMAMCL and NGP had the highest sensitivity. The possible reason for this phenomenon maybe that BE2 cells have MYCN amplification, P53 mutation and 1pLOH (loss of heterozygosity at chromosome 1p), while NGP cells only have MYCN amplification. Furthermore, our findings suggest that tumor cells are more sensitive to DMAMCL than normal cells, also supporting the clinical application of DMAMCL to treat NB. Recent study showed that spatial learning and memory ability in mice was significantly improved by chronic median dose DMAMCL (25 mg/kg) treatment [32]. It proved that DMAMCL may not only play a role in inhibiting NB tumors, but also improve the cognitive function of mice within a certain concentration range, indicating that DMAMCL has fewer toxic effects and supports the clinical application of DMAMCL.

Etoposide and cisplatin are the first-line chemotherapy drugs for NB in clinic. Etoposide is a topoisomerase inhibitor and causes DNA strands break, which causes errors in DNA synthesis and promotes cell death. Cisplatin mainly causes cell death by affecting the cell cycle and interferes with DNA replication. In this study, we aimed to select chemotherapeutic drugs with different mechanisms and detect the combination effects with DMAMCL in vitro and in vivo*.* For instance, DMAMCL has been found to potently sensitize HCC cells to various clinical chemotherapeutic agents (gemcitabine, paclitaxel, doxorubicin, cisplatin) [27] and reverse resistance to cisplatin and tamoxifen in breast cancer [12, 13]. Moreover, a study found that combining DMAMCL with vincristine or epirubicin increases cell death and tumor growth inhibition in vivo in rhabdomyosarcoma (RMS) [33]. Consistently, our study found that DMAMCL exerts significant synergistic effects with etoposide or cisplatin in NB cells in vitro and notably increased antitumor effects when combined with etoposide in the xenograft mouse model. More importantly, the combination of DMAMCL with etoposide significantly improved the survival of the mice without affecting body weight (Additional file 4: Fig. S4B, C), suggesting that DMAMCL can be combined with chemotherapeutic agents to treat NB.

Metabolic reprogramming is widely recognized as a characteristic hallmark of cancer cells that contributes toward tumor development [34–37]. Unlike normal cells, cancer cells primarily rely on aerobic glycolysis for glucose metabolism, even under normoxic conditions [38–40]. Recent studies have indicated the importance of exploring the mechanisms underlying the regulation of aerobic glycolysis in NB to develop effective therapeutic strategies. Gan et al*.* reported that 3-bromopyruvate (3-BrPA), a hexokinase (HK)-II inhibitor, combined with rapamycin, synergistically suppressed aerobic glycolysis in NB cells [41]. Song et al*.* reported that therapeutic targeting of the HNF4A-AS1/hnRNPU/CTCF axis inhibited aerobic glycolysis and NB progression [42]. Fang et al. determined that therapeutic targeting of the YY1/MZF1 axis by MZF1-uPEP inhibited aerobic glycolysis of NB cells [43]. Another study by Fang et al*.* found that valproic acid (VPA), an established histone deacetylase inhibitor, suppressed aerobic glycolysis and tumor progression of NB, indicating a novel therapeutic strategy for NB [44]. Importantly, DMAMCL reportedly plays a role in inhibiting aerobic glycolysis. Li et al*.* and Guo et al*.* found that DMAMCL decreased lactate production, a surrogate indicator of glycolysis, in leukemia and glioma, suggesting that DMAMCL inhibits aerobic glycolysis [11, 15]. In this study, we directly measured the real time ECAR by Seahorse XFe96, which is a standard and comprehensive method for assessing key parameters of glycolytic flux. We also found that glucose consumption, lactate excretion and ATP production were all decreased after DMAMCL treatment in NB cells, confirming that DMAMCL suppresses aerobic glycolysis in NB cells.

Recent studies have found that the rate-limiting glycolysis enzyme, PKM2, is a key target for the DMAMCL-induced inhibition of cell proliferation in leukemia and glioma; however, these studies found no significant differences in PFKL or HK2 mRNA expression [11, 15]. Here, we detected decreased PFKL, PKM2, and HK2 protein expression in vitro after DMAMCL treatment, with PFKL protein expression decreasing much earlier than PKM2 or HK2 in both NGP and BE2 cells. Therefore, the regulation of aerobic glycolysis may be a potential mechanism underlying the effects of DMAMCL, which modulates various different metabolic enzymes. PFKL has also been shown to play an essential role in tumorigenesis [45–48], with its up-regulation promoting tumor cell proliferation and metabolic reprogramming in various cancers, such as glioma and breast cancer [49–52]. Although PFKL overexpression did not significantly increase NB cell survival in this study, it remarkably attenuated DMAMCL-induced NB cell death. Meanwhile, downregulating PFKL promoted NB cell death, consistent with a previous study in which PFKL knockdown significantly suppressed the progression of oral cancer [53]. Together, these reports demonstrate that PFKL plays a critical role in the antitumor effects induced by DMAMCL in NB. Furthermore, the activity of PFKL is higher when its tetramer structure is maintained, whereas its activity decreases when PFKL is allosterically regulated to become an oligomeric dimer [54]. We hypothesize that DMAMCL may regulate PFKL by changing the protein structure of PFKL or by undergoing special protein post-translational modifications. These hypotheses will be investigated in future research.

The mechanisms underlying the antitumor effects of DMAMCL have been reported in various cancers. In leukemia, DMAMCL induces cell death by inhibiting the NF-κB pathway [28], whereas DMAMCL induces apoptosis in osteosarcoma by activating caspase-dependent pathways [55] and inactivates the PI3K/Akt pathway in HCC [27]. We also evaluated p-Akt and NF-κB expression after DMAMCL treatment in this study, but didn’t detect a decrease in p-Akt and NF-κB expression in NB cells (data not shown), suggesting that DMAMCL may induce cell death via various different mechanisms. Although we found that up-regulating PFKL blocked DMAMCL-induced cell death in NB cells, this blockage was only partial, potentially because of a low cell transfection efficiency (50–70%) causing the role of PFKL to be underestimated. Another reason for the partial role of PFKL could be that other molecules also mediate the effects of DMAMCL. Li et al. reported that DMAMCL selectively represses acute myelogenous leukemia stem cells [9]; however, no studies have yet investigated whether DMAMCL selectively acts against NB stem cells. Therefore, our future studies will explore other mechanisms of DMAMCL and its role in NB stem cells using high-throughput screening.

However, there are still some limitations in the present study, which warrant further investigation. In addition to affecting the glycolytic capacity and the expression of PFKL in NB, whether other metabolic processes, or the key genes or pathways are involved in the inhibitory effect of DMAMCL on NB were not studied. Therefore, our following study will aim to explore other mechanisms of DMAMCL in NB treatment through the integration of metabolomic and transcriptomic data.

## Conclusions

In conclusion, the results of this study suggest that DMAMCL exerts antitumor effects in NB both in vitro and in vivo by suppressing aerobic glycolysis and that PFKL could be a potential target of DMAMCL in NB (Additional file 5: Fig. S5). Therefore, our study supports the future clinical evaluation of DMAMCL in NB treatment and proposes that targeting PFKL could be an additional treatment avenue for metabolic modulation in NB.

## Supplementary Information

**Additional file 1: Figure S1.**  The scheme of the in vivo experiment.

**Additional file 2: Figure S2.** Etoposide and cisplatin dose-dependently inhibited NGP and BE2 cells. NGP and BE2 cells were treated with different concentrations of etoposide or cisplatin for 72 h. (A, B) Survival of NGP and BE2 cells treated with different concentrations of (A) etoposide and (B) cisplatin detected using a CCK-8 assay.

**Additional file 3: Figure S3.**  DMAMCL has no significant effect on the glycolysis of NIH3T3 cells were treated with different DMAMCL concentrations for 24h, ECAR was measured by adding glucose, oligomycin (ATP synthase inhibitors), and 2-deoxy-D-glucose (2-DG, hexokinase inhibitor) in turn to reflect the glycolysis rate including the glycolytic function, basal glycolysis, and glycolytic capacity. (A) Glycolytic function, (B) Basal glycolysis and Glycolytic capacity of NIH3T3 cells. ns (not significant): control vs DMAMCL treatment, * p<0.05.

**Additional file 4: Figure S4.**  DMAMCL and etoposide exerted no obvious effect on body weight in mice bearing NGP and BE2 tumors. (A) Body weight of mice bearing NGP and BE2 tumors treated with DMAMCL (75 mg/kg or 100 mg/kg). ns (not significant): control vs DMAMCL (75 mg/kg or 100 mg/kg), * p<0.05. (B) Body weight of mice bearing NGP tumors treated with DMAMCL (75 mg/kg) and etoposide alone or in combination. (C) Body weight of mice bearing BE2 tumors treated with DMAMCL (75 mg/kg or 100 mg/kg) and etoposide alone or in combination. ns (not significant): DMAMCL / Etoposide vs DMAMCL +Etoposide, * p<0.05.

**Additional file 5: Figure S5.**  The hypothesis figure.

## Data Availability

The datasets used and analyzed during this study are available from the corresponding author upon reasonable request.

## Abbreviations

NB: Neuroblastoma
DMAMCL: Dimethylaminomicheliolide
PFKL: Liver type of phosphofructokinase-1
MCL: Micheliolide
F6P: Fructose 6-phosphate
F-1,6-BP: Fructose 1,6-bisphosphate
PFK-1: Phosphofructokinase -1
HK2: Hexokinase 2
PKM2: Pyruvate kinase M2
