# Supplementary figures and images for: Antitumor effects of the small molecule DMAMCL in neuroblastoma via suppressing aerobic glycolysis and targeting PFKL
Source: Cancer Cell Int. 2021 Nov 24;21:619. doi: 10.1186/s12935-021-02330-y (PMC8613996; doi:10.1186/s12935-021-02330-y)

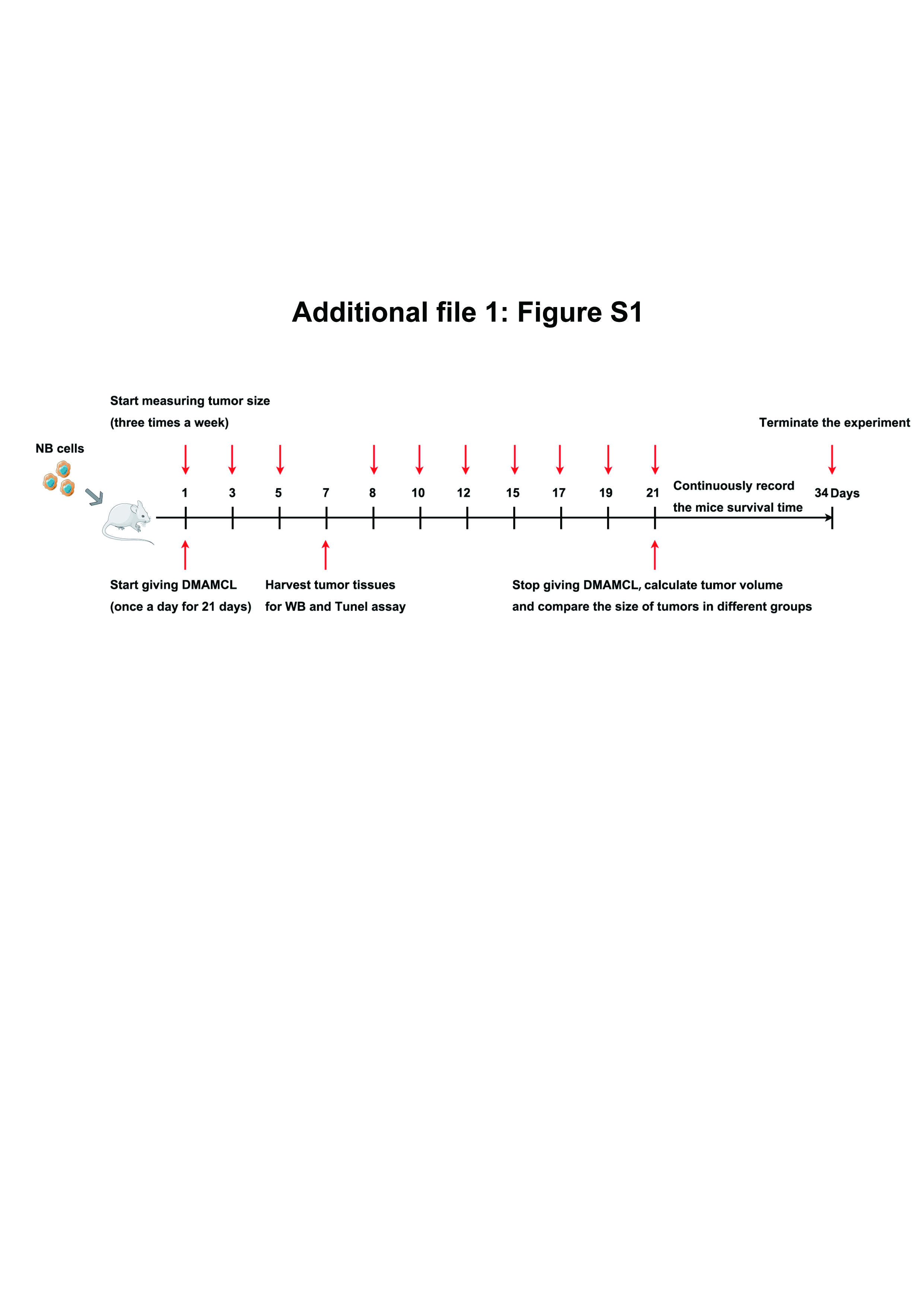

Supplement: Supplementary file 1 — Additional file 1: Figure S1. The scheme of the in vivo experiment. [file 12935_2021_2330_MOESM1_ESM.tif]

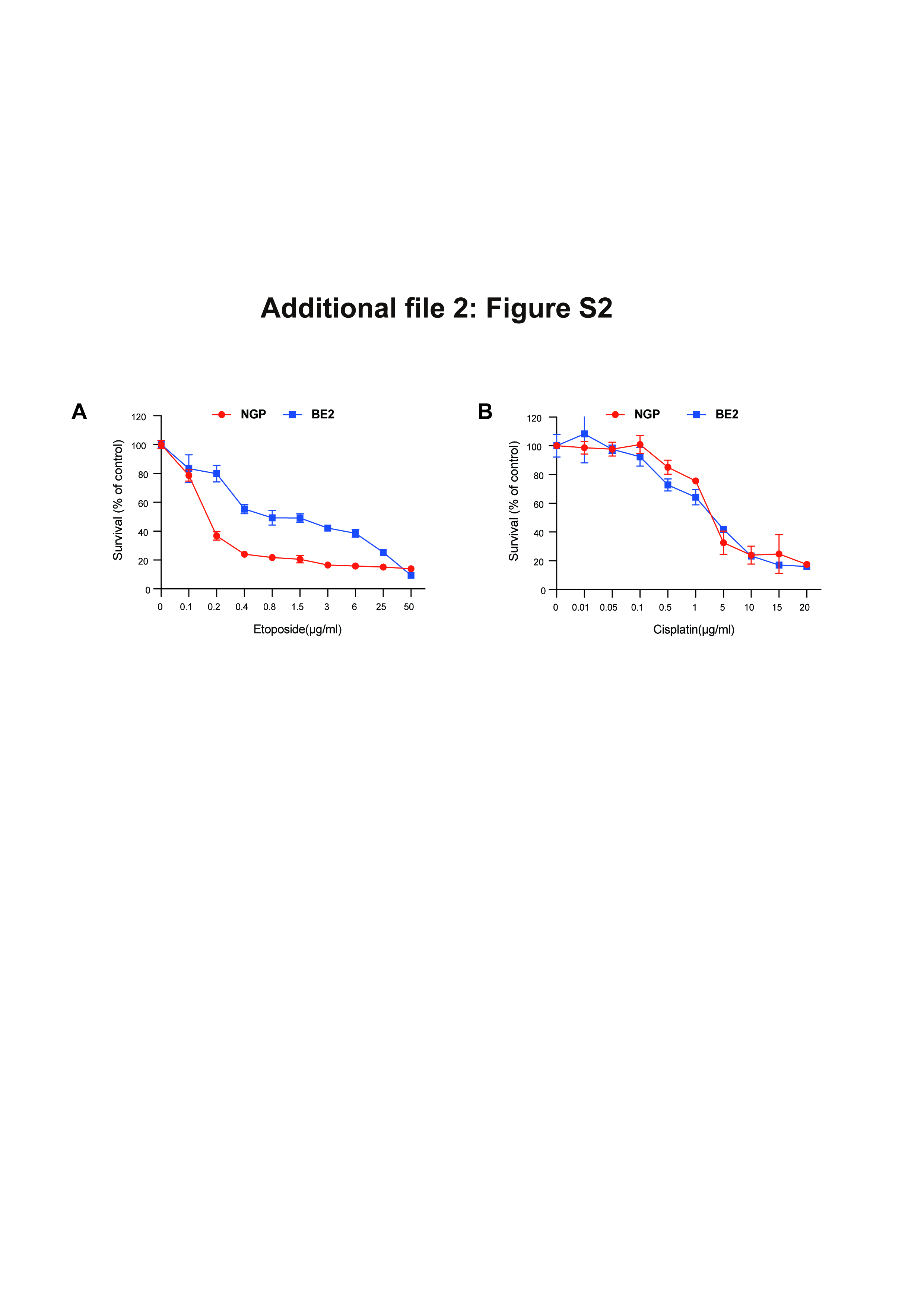

Supplement: Supplementary file 2 — Additional file 2: Figure S2. Etoposide and cisplatin dose-dependently inhibited NGP and BE2 cells. NGP and BE2 cells were treated with different concentrations of etoposide or cisplatin for 72 h. (A, B) Survival of NGP and BE2 cells treated with different concentrations of (A) etoposide and (B) cisplatin detected using a CCK-8 assay. [file 12935_2021_2330_MOESM2_ESM.tif]

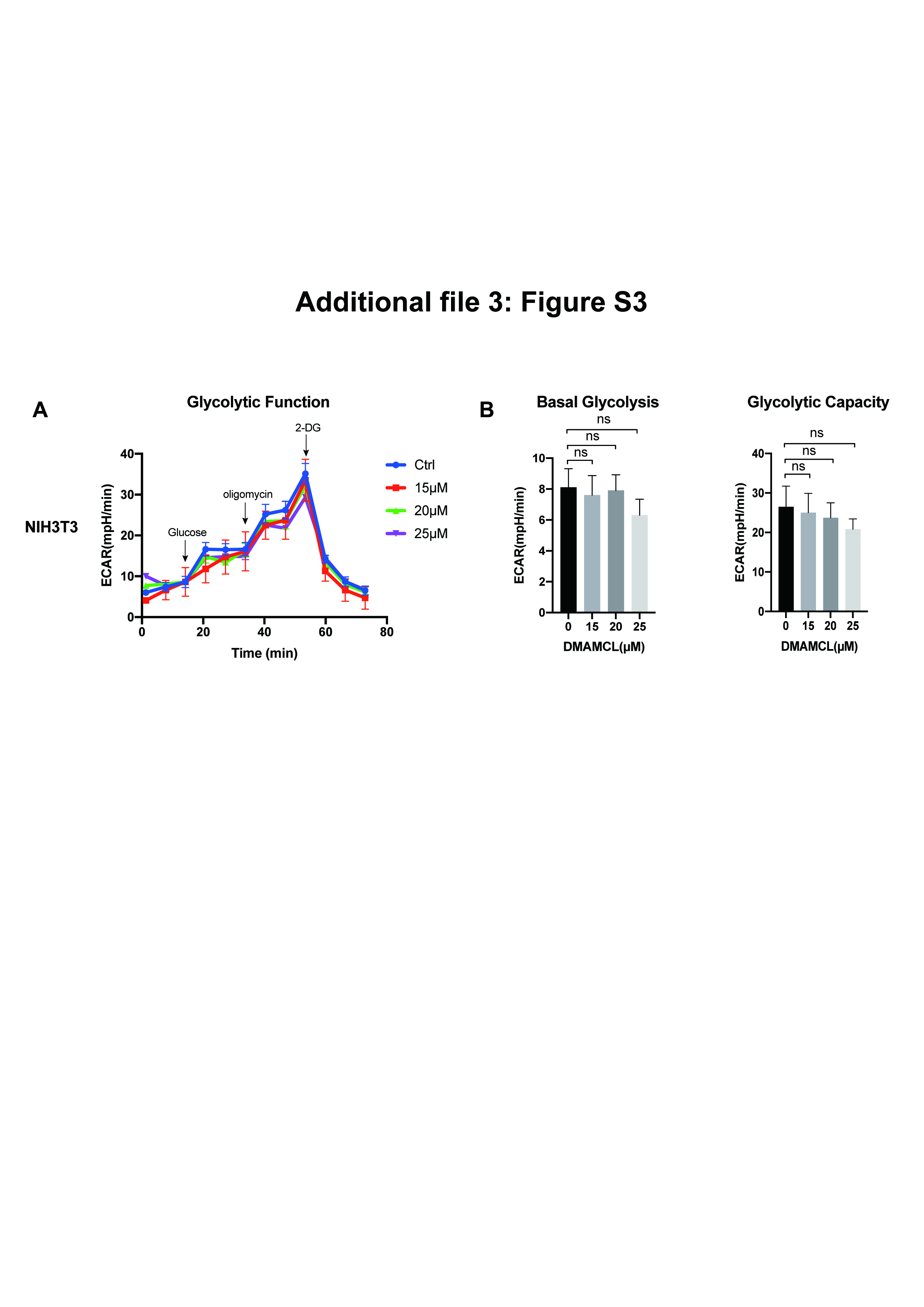

Supplement: Supplementary file 3 — Additional file 3: Figure S3. DMAMCL has no significant effect on the glycolysis of NIH3T3 cells were treated with different DMAMCL concentrations for 24h, ECAR was measured by adding glucose, oligomycin (ATP synthase inhibitors), and 2-deoxy-D-glucose (2-DG, hexokinase inhibitor) in turn to reflect the glycolysis rate including the glycolytic function, basal glycolysis, and glycolytic capacity. (A) Glycolytic function, (B) Basal glycolysis and Glycolytic capacity of NIH3T3 cells. ns (not significant): control vs DMAMCL treatment, * p<0.05. [file 12935_2021_2330_MOESM3_ESM.tif]

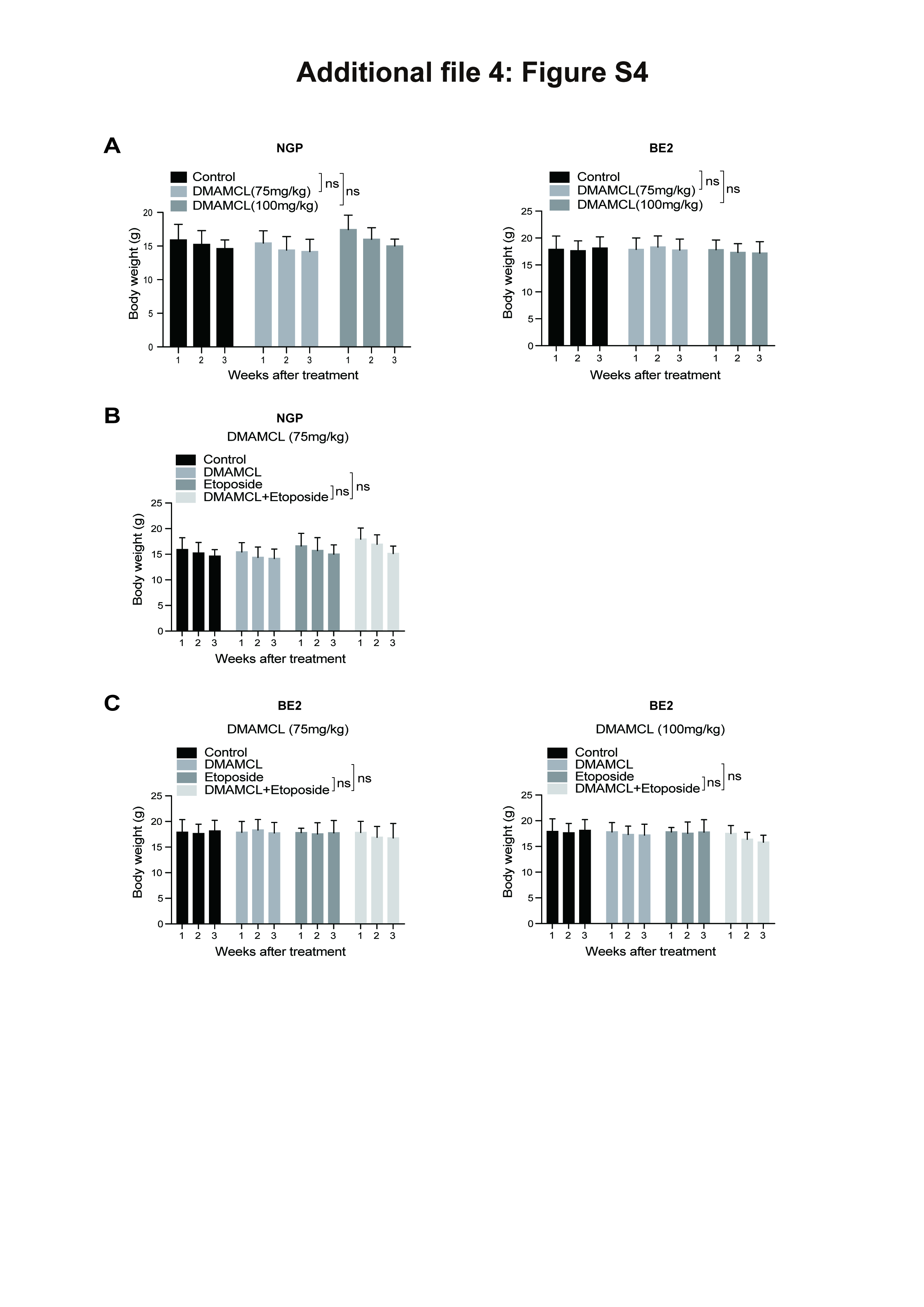

Supplement: Supplementary file 4 — Additional file 4: Figure S4. DMAMCL and etoposide exerted no obvious effect on body weight in mice bearing NGP and BE2 tumors. (A) Body weight of mice bearing NGP and BE2 tumors treated with DMAMCL (75 mg/kg or 100 mg/kg). ns (not significant): control vs DMAMCL (75 mg/kg or 100 mg/kg), * p<0.05. (B) Body weight of mice bearing NGP tumors treated with DMAMCL (75 mg/kg) and etoposide alone or in combination. (C) Body weight of mice bearing BE2 tumors treated with DMAMCL (75 mg/kg or 100 mg/kg) and etoposide alone or in combination. ns (not significant): DMAMCL / Etoposide vs DMAMCL +Etoposide, * p<0.05. [file 12935_2021_2330_MOESM4_ESM.tif]

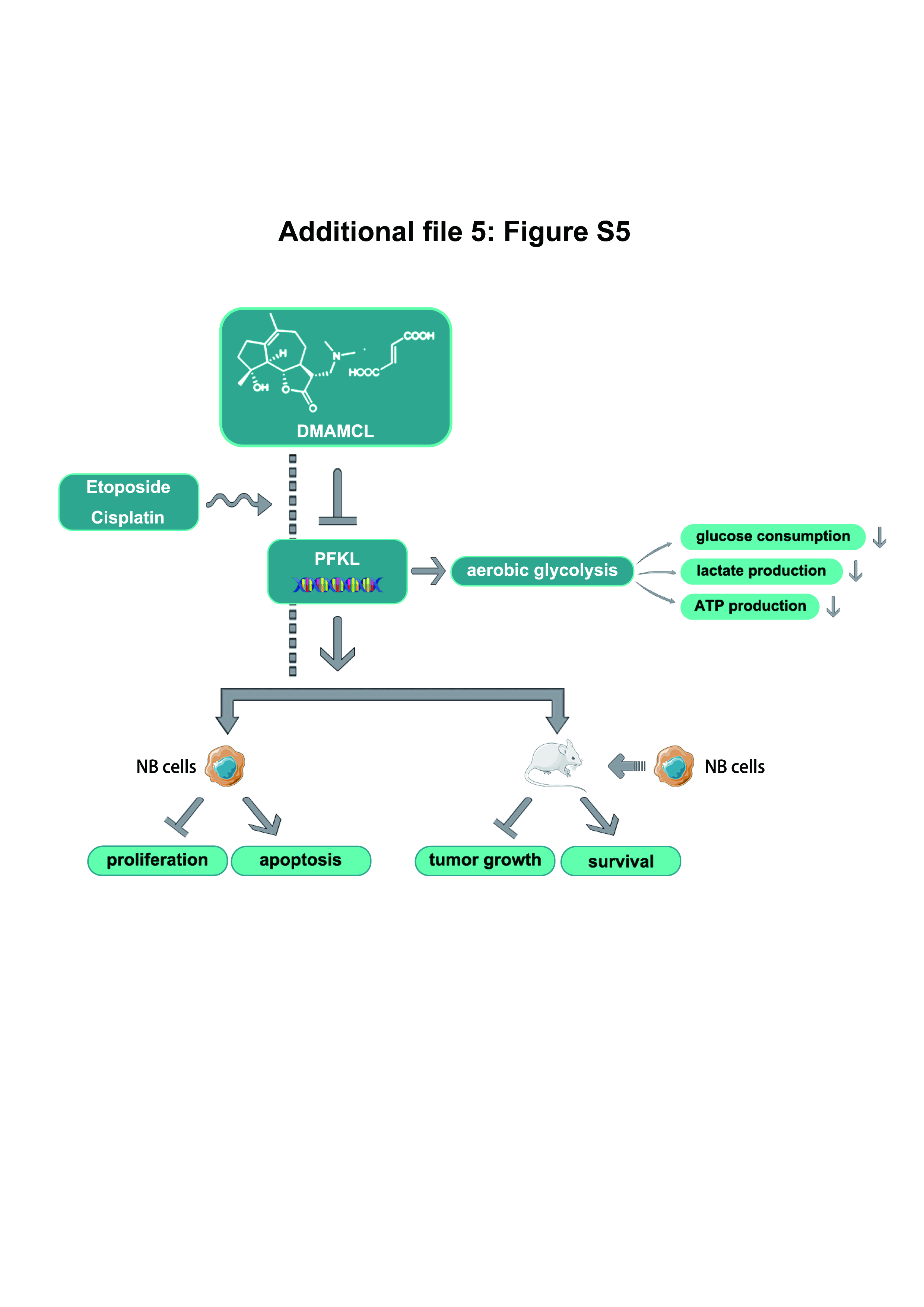

Supplement: Supplementary file 5 — Additional file 5: Figure S5. The hypothesis figure. [file 12935_2021_2330_MOESM5_ESM.tif]
